# Advances in biomimetic stimuli responsive soft grippers

**DOI:** 10.1186/s40580-019-0191-4

**Published:** 2019-07-01

**Authors:** ChangKyu Yoon

**Affiliations:** Department of Mechanical Systems Engineering, Sookmyung Women’s University, Seoul, 04310 Republic of Korea

**Keywords:** Soft actuators, Intelligent systems, Self-folding, Soft robots, Bio-MEMS

## Abstract

A variety of biomimetic stimuli-responsive soft grippers that can be utilized as intelligent actuators, sensors, or biomedical tools have been developed. This review covers stimuli-responsive materials, fabrication methods, and applications of soft grippers. This review specifically describes the current research progress in stimuli-responsive grippers composed of *N*-isopropylacrylamide hydrogel, thermal and light-responding liquid crystalline and/or pneumatic-driven shape-morphing elastomers. Furthermore, this article provides a brief overview of high-throughput assembly methods, such as photolithography and direct printing approaches, to create stimuli-responsive soft grippers. This review primarily focuses on stimuli-responsive soft gripping robots that can be utilized as tethered/untethered multiscale smart soft actuators, manipulators, or biomedical devices.

## Introduction

Inspired by the shape change of biological systems, such as *Rhododendron* [1] and *Dionaea muscipula* (Venus flytrap) leaves [2, 3], biomimetic shape-morphing soft robots have been extensively proposed by utilizing stimuli-responsive hydrogels, polymer, or their hybrid combination [4–6]. The stimuli responsive materials and their architectures can be transformed into three-dimensional (3D) self-assembled, -curved, or -folded structures in response to external triggers without any manual control [4]. In particular, newly emerged biomimetic stimuli-responsive soft gripping systems have been highlighted because of their promising applications in smart actuators, flexible electronics, biosensors, micro/nanomanipulators, smart medicine, and surgery [7–30].

In engineering biomimetic soft grippers, hydrogels, and polymer are attractive materials for the following reasons: first, polymer or hydrogels exhibit moduli ranges (~ KPa) similar to those of biological tissues and organs [31]; and second, polymer or hydrogels can swell by several orders of magnitudes in volume in response to external stimuli [32, 33]. This swelling/de-swelling mechanism can yield large deformations that enable self-actuation without any tethered external power sources. Multilayer thin-film fabrication or direct printing is widely used to create biomimetic soft grippers because multilayer structures can achieve spontaneous 3D curving, wrinkling, or folding shapes using different swelling behaviors between layers [18]. Furthermore, diverse engineering methodologies, such as photolithography, 3D printing, direct contact mode molding, or micro- and nano-imprinting, can be utilized to create stimuli-responsive soft grippers for advanced functional biomimetic actuators, drug delivery capsules, tiny biopsy tools, or valves for lab-on-a-chip applications [34].

A broad discussion of stimuli-responsive materials and applications [35–40] and a variety of shape-changeable soft robotic systems have been reviewed for a more comprehensive analysis on the recent advances in soft robotics [31, 41–45]. This review primarily focuses on the recent progress of stimuli-responsive soft grippers in terms of material designs, fabrication methods, and applications. First, we summarize the stimuli-responsive materials, including *N*-isopropylacrylamide (NIPAM)-based hydrogels, liquid crystalline networks, and elastomers. Next, we present engineering techniques, such as photolithography and direct printing methods, to create soft gripping devices by organizing the stimuli-responsive materials. We then give an overview of the applications of the stimuli-responsive soft grippers in a number of fields, including soft machines, biological medicine, and surgical tools. Finally, we discuss the current open challenges and possible new fields of interest for these stimuli-responsive soft grippers.

## Material selection

### *N*-isopropylacrylamide (NIPAM)-based stimuli-responsive hydrogels

**Fig. 1:**
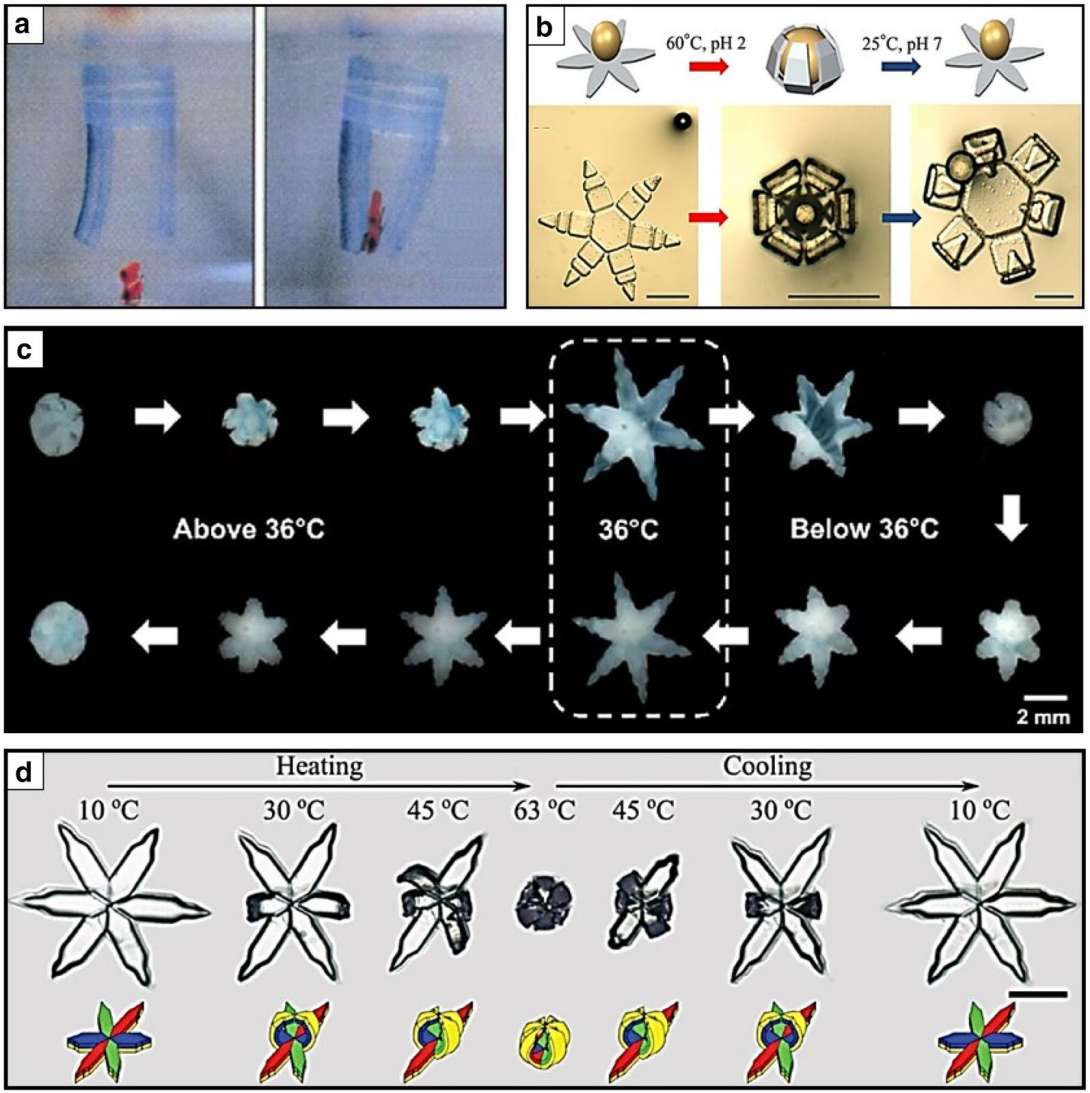
Biomimetic soft gripping robots composed of stimuli responsive hydrogels, polymer, or hybrid combination of them. **a** *N*-isopropylacrylamide (NIPAM)-modulated thermal responsive bigel stripped grippers (reproduced with permission [17]. Copyright 1995, AAAS). **b** Poly*(N*-isopropylacrylamide-acrylic acid)(pNIPAM-AAc) soft gripper that reversibly actuates when triggered by temperature or pH (reproduced with permission [13]. Copyright 2014, IOP Publishing). **c** Thermally responsive self-folding bilayer soft gripper that closes and opens reversibly when passing by LCST at 36 °C (reproduced with permission [9]. Copyright 2015 American Chemical Society). **d** Reversible four-state shape changes of soft grippers during heating and cooling process (reproduced with permission [26]. Copyright 2018 Wiley–VCH)

Stimuli-responsive materials refer to a new class of materials that change their chemical and physical properties in response to external stimuli, such as heat (electro-, photo-thermal), pH, magnetic fields, light, and biochemical enzymes [4, 46–48]. Most stimuli-responsive materials have generally been synthesized by combining hydrophilic (e.g., amide and carboxyl) and hydrophobic (i.e., methyl, ethyl, and propyl) groups in a single gel network [47]. These gel-network designs demonstrate a sharp critical transition point known as the lower critical solution temperature (LCST) that exhibits unique property changes in a gel system [46]. Below the LCST, these gel systems exhibit hydrophilic properties because they absorb water, while above the LCST, the hydrophobic properties become dominant and result in water desorption. NIPAM is one of the important stimuli-responsive LCST hydrogels [49]. Above the LCST, NIPAM-based hydrogels exhibit hydrophobicity (de-swollen) and undergoes hydrophilicity (swollen) below the LCST between 32 and 36 °C [34, 46, 50]. The swelling/de-swelling mechanism near the LCST can generate the shape changes of NIPAM-based hydrogels when exposed to external stimuli. Notably, thermoresponsive NIPAM-based hydrogels are extensively utilized because of the easy access to the heat source [9, 12, 13, 17, 18, 29, 51–62]. The first thermal-responsive NIPAM-modulated soft grippers were proposed by Hu et al. in 1995 (Fig. 1a) [17]. They fabricated two bigel strips that could grip and release an object by temperature change. The acrylamide (PAAM)/NIPAM strips could open at room temperature and close at 35 °C reversibly because of the large amount of strain caused by the swelling/deswelling of the thermoresponsive NIPAM layer. These swelling changes in a bigel strip system can generate shape changes to grip a target. They have initially introduced the potentials of mechanized small-scale soft robots using thermoresponsive NIPAM-based hydrogels.

Meanwhile, Gracias et al. proposed several shape transformable stimuli responsive grippers using pNIPAM-based hydrogels [9, 10, 13, 63, 64]. They developed a monolayer of pNIPAM-co-acrylic acid (AAc) gripper that could actuate by using a thin crosslinking gradient along the thickness in hinges (Fig. 1b) [13]. However, the thin-monolayer soft gripper did not have enough force to grip an object because of its softness. Accordingly, they developed bilayer geometric grippers composed of stiff (16 MPa) segments polypropylene fumarate (PPF) and thermally responsive low shear modulus (162 kPa) pNIPAM-AAc (Fig. 1c) [9]. They specifically validated that these bilayer grippers possess sufficient strength to excise a cell from tissue clumps. Furthermore, they demonstrated remotely guided soft grippers using magnetic fields by embedding iron oxide (Fe_2_O_3_) nanoparticles. Furthermore, they recently reported multistate shape-changing bilayer soft grippers composed of poly (oligo [ethylene glycol] methyl ether methacrylate) (POEGMA) (Fig. 1d) [26]. The hand-shaped grippers were composed of POEGMA-based multi domains that exhibited different LCSTs and volume transition temperature such that these grippers reversibly underwent multistate folding and unfolding according to several heating and cooling cycles.

**Fig. 2:**
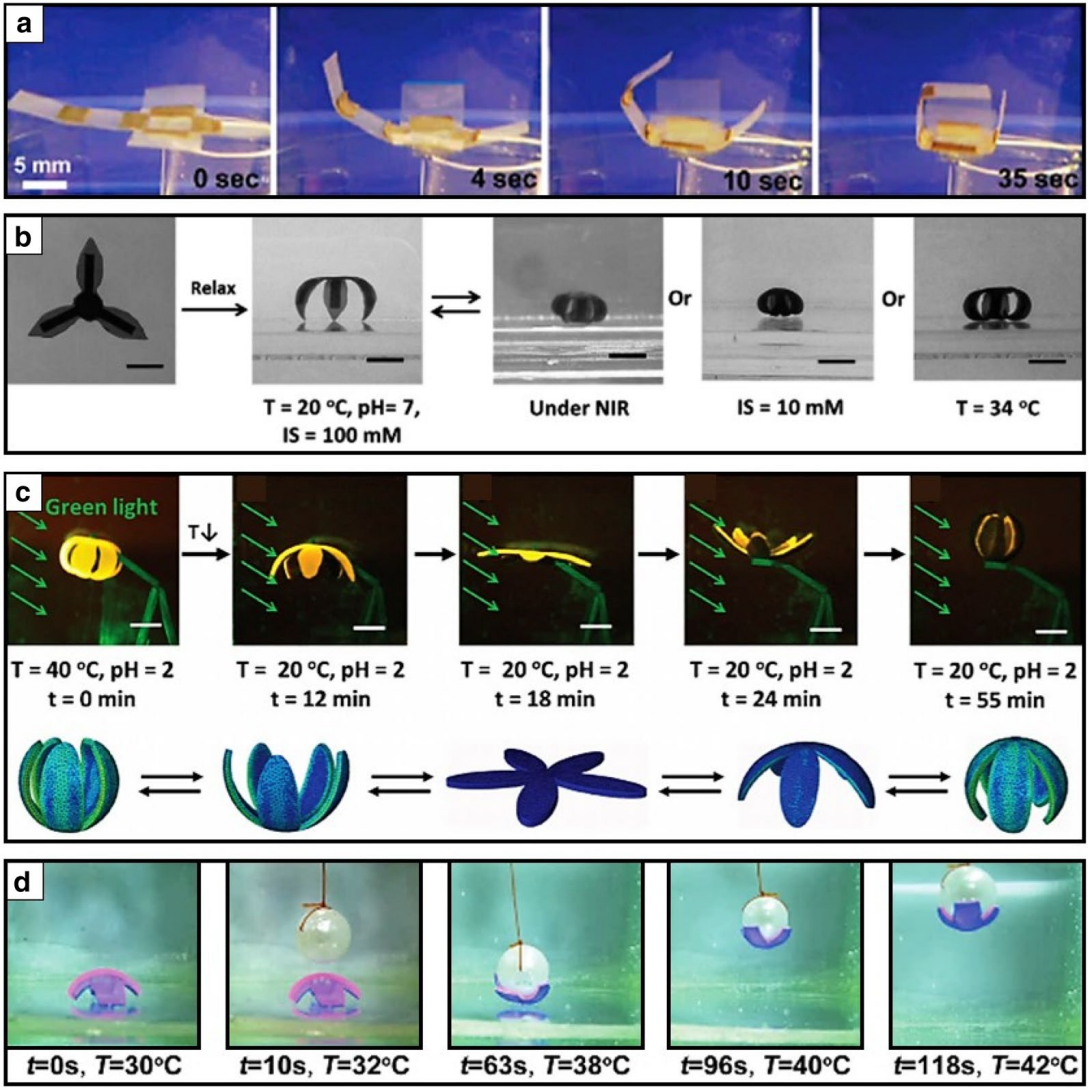
Biomimetic stimuli responsive soft grippers composed of poly *N*-isopropylacrylamide (pNIPAM) based hydrogels hybridized with nanoparticles. **a** Programmable folding cube composed of single-walled carbon nanotube (SWNT)-pNIPAM and low-density polyethylene (LDPE) bilayer that actuates reversibly in water (reproduced with permission [66]. Copyright 2011, American Chemical Society). **b** Multi near-infrared light (NIR), ionic strength (IS), and temperature change responsive soft gripper composed of graphene oxide (GO)-pNIPAM and pNIPAM-poly(methylacrylic acid)(PMAA) bilayer (reproduced with permission [65]. Copyright 2016, Wiley–VCH). **c** Thermoresponsive biomimetic flower shaped fluorescent color displaying soft gripper composed of graphene oxide (GO)-pNIPAM and pH responsive perylene bisimide-functionalized hyperbranched polyethylenimine (PBI-HPEI) hybrid bilayer (reproduced with permission [68]. Copyright 2017, Wiley–VCH). **d** Temperature-controlled pNIPAM/pNIPAM-co-clay nanocomposite bilayer hydrogel gripper that grips a moving pearl (reproduced with permission [22]. Copyright 2015, Wiley–VCH)

In addition, pNIPAM hydrogels have been combined with nanoparticles to realize multi-responsive and highly sensitive stimuli-responsive soft grippers [22, 23, 27, 29, 65, 66]. The nanoparticles play an important role in optical-to-thermal-energy-transferring systems as an embedded form inside a hydrogel. Zhang et al. proposed a reversible, optically, and thermally responsive actuator composed of pNIPAM/single-walled carbon nanotube (SWCN) composites (Fig. 2a) [66]. They observed an ultrafast near-infrared optical response in SWCN/pNIPAM hydrogel actuators under laser excitation. The actuation of these hydrogel systems was caused by the strong light absorptions of the nanotube where the amount of absorptions was controlled by the nanotube loading ratio. In addition, Chen et al. recently proposed graphene oxide sheet (GOs)-embedded NIPAM-based soft actuators that respond to multi-environmental heat, pH, light, and ionic strength triggers [65, 67, 68]. They developed soft grippers composed of GO-pNIPAM with pNIPAM-poly(methylacrylic acid)(PMAA) bilayers that simultaneously responded to near-infrared light, ionic strength, and temperature change (Fig. 2b) [65]. Furthermore, they recently presented an anisotropic bilayer hydrogel actuator with an on–off switchable fluorescent color-changing function composed of GO-pNIPAM and pH responsive perylene bisimide-functionalized hyperbranched polyethylenimine (PBI-HPEI) hybrid bilayer structures (Fig. 2c) [68]. In particular, the shape deformation of a biomimetic flower-shaped soft gripper was provoked under green light irradiation when GO-pNIPAM and PBI-HPEI layers were exposed to the on–off switch of the thermal- and pH-responsive fluorescence. Regarding the formation of smart stimuli-responsive soft gripping systems, Yao et al. also suggested pNIPAM/clay nanocomposite hydrogel grippers that exhibit rapid, reversible, and repeatable actuations (Fig. 2d) [22, 23]. They proposed nanoclay-crosslinked pNIPAM hydrogel structures exhibiting a high thermal response that can be utilized as a manipulator. In addition, Shi et al. proposed hand-shaped grippers that could actuate by transferring light to thermal energy through embedding an energy transformation agent of gold nanoparticles in pNIPAM hydrogel [29].

Besides the hybrid nanoparticles with pNIPAM hydrogels, Ko et al. have developed low voltage driven electro-thermally shape changeable soft grippers composed of the silver nanowire (Ag NW) deposited low-density polyethylene (LDPE) and thermochromic ink deposited polyvinylchloride (PVC) bilayer [69]. Particularly, they proposed a variety of biomimetic color changeable flower- or tendril-shaped self-bending, -rolling, or -twisting soft actuators that demonstrated long-term stability of actuation under more than 10,000 cycles of heating and cooling conditions. Furthermore, their Ag NW percolation network heater could generate sufficient heat to obtain large deformation (curvature up to 2.5 cm^−1^ at 40 °C) at low voltages compared to other high voltage driven electroactive polymer actuators [70]. These unique mechanical properties coupled with thermally-driven color shifting characteristics of soft electro-thermal actuators (ETA) have opened up another new possible field of intelligent camouflageable soft robots.

### Liquid crystalline material-based stimuli-responsive hydrogels

**Fig. 3:**
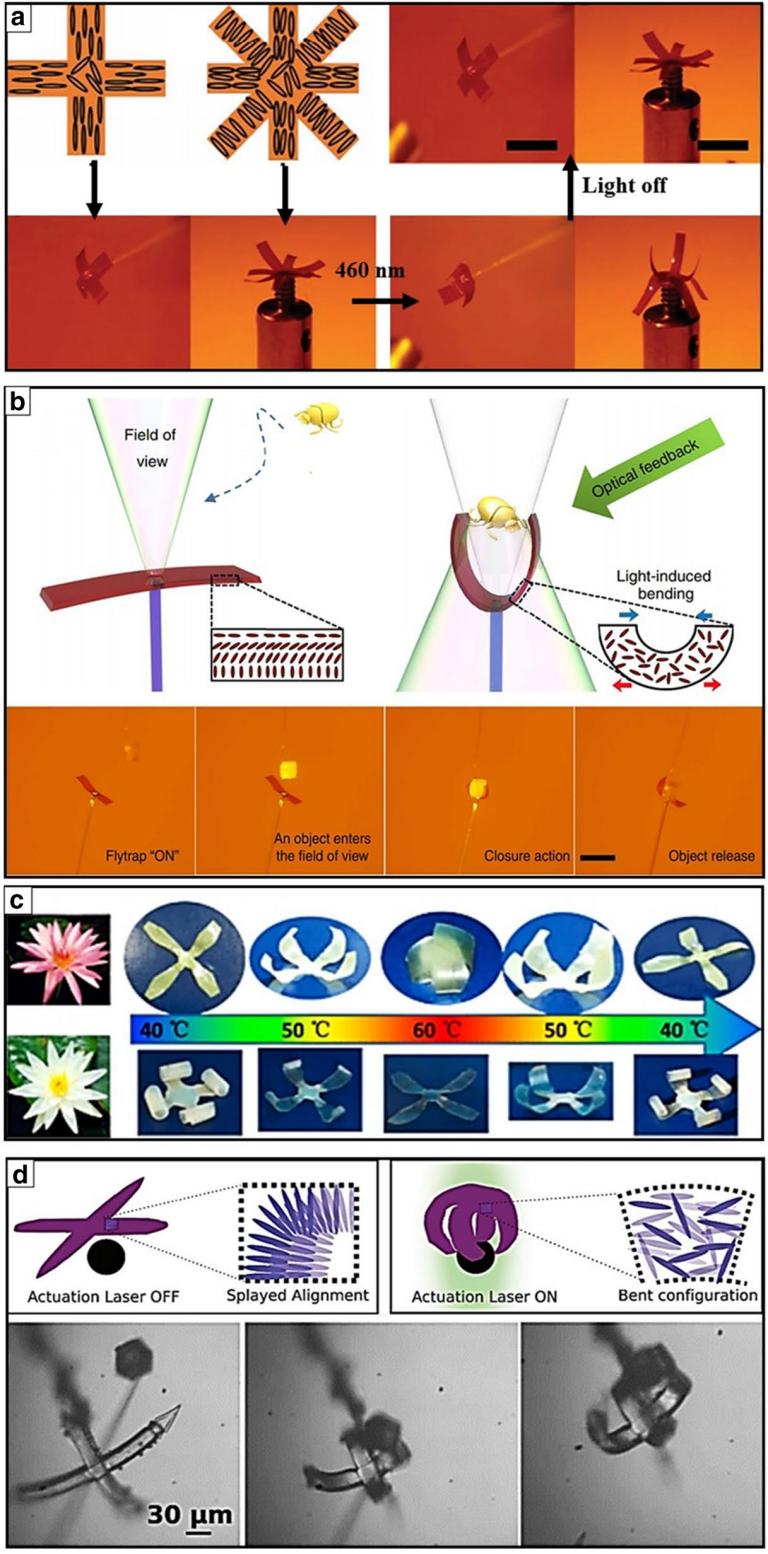
Photothermal responsive liquid crystalline networks (LCNs) and liquid crystalline elastomers (LCEs) soft grippers. **a** Photothermal actuation of 4- or 8-armed soft grippers when exposed to 460 nm illumination that are composed of splay- or − 90° twisted nematic alignment patterns in a liquid crystal polymer networks film (reproduced with permission [75]. Copyright 2017, Wiley–VCH). **b** Flytrap mimetic light responsive self-folding liquid crystal elastomers (LCEs) gripper that captures an object according to light illumination intensity feedback (reproduced with permission [76]. Adapted with permission under the terms of the Creative Commons Attribution Non Commercial License 4.0 license. Copyright 2017, The Authors). **c** Water Lily flower mimicked thermoresponsive soft liquid crystal networks (LCNs) grippers that can open and close via induced smetic–nematic phase transition in LCNs according to heating and cooling process (reproduced with permission [7]. Copyright 2018, American Chemical Society). **d** Light driven actuation of LCNs soft grippers controlled by the mesogen alignment change (reproduced with permission [28]. Copyright 2017, Wiley–VCH)

Liquid crystalline materials, such as liquid crystalline elastomers (LCEs) and liquid crystalline networks (LCNs), have been studied as one of the promising systems that display shape morphing when triggered by external stimuli sources [71]. LCEs and LCNs can generally be categorized according to the glass transition temperature (T_g_) and mechanical properties [72]. LCEs exhibit a T_g_ below room temperature in the order of MPa, whereas LCNs possess higher T_g_ with elastic modulus [73]. LCN and LCE hybrids can specifically exhibit shape memory behaviors by tuning the alignment of molecules (twisted nematic and splay configuration) and crosslinking degree, which results in reversible shape changes, such as bending, twisting, rotating, or folding, after applying external stimuli, such as heat, light, and humidity [74]. Responsive liquid crystalline-based smart soft gripping robots have been developed by exploring the photo-aligning properties of liquid crystalline materials. Accordingly, Priimagi et al. proposed light-responsive four- or eight-legged soft grippers that could display smart shape deformation by tuning the photoalignment of programmable molecular orientation in LCNs (Fig. 3a) [75]. They adapted a MEMS-inspired scanning mirror assembled laser project to program the alignment of liquid crystals within a monolithic LCN thin film in an efficient and easy fabrication manner. They specifically programmed splay-aligned four-armed or 90°-twisted nematic-aligned eight-armed grippers that featured photothermal actuation by switching on/off a 460 nm optical light source. They also proposed a highly sensitive light-driven biomimetic artificial flytrap composed of thin LCEs (Fig. 3b) [76]. They developed self-recognitive autonomous light-driven LCE gripping systems based on optical feedback as a function of illumination laser intensity. Particularly, they adapted an optical fiber to deliver light energy to deform LCE micro grippers when triggered by scattered light. They demonstrated the possibility of autonomous and intelligent light-power driven biomimetic micro robots. Meanwhile, Zhao et al. also introduced flower-shaped LCN soft grippers to mimic intelligent biological systems (Fig. 3c) [7]. They proposed multitemperature responsive actuators controlled by thermal responsive smetic-nematic phase transition in LCNs during the reversible cycles of heating and cooling. In addition, the Wiersma group presented several liquid crystalline material-based stimuli-responsive actuating systems with a comprehensive review of the LCNs and the LCEs and their applications [28, 72, 74]. In particular, their thermal and light-responsive soft microscale grippers composed of LCNs and LCEs displayed the optical-driven reversible actuation of folding and unfolding (Fig. 3d) [28]. According to the on–off process of light, the microgripper was flat with a splayed alignment (on-mode) and folded with a bent anisotropic configuration of molecules (off-mode).

## Methodology: Photolithography or 3D printing

**Fig. 4:**
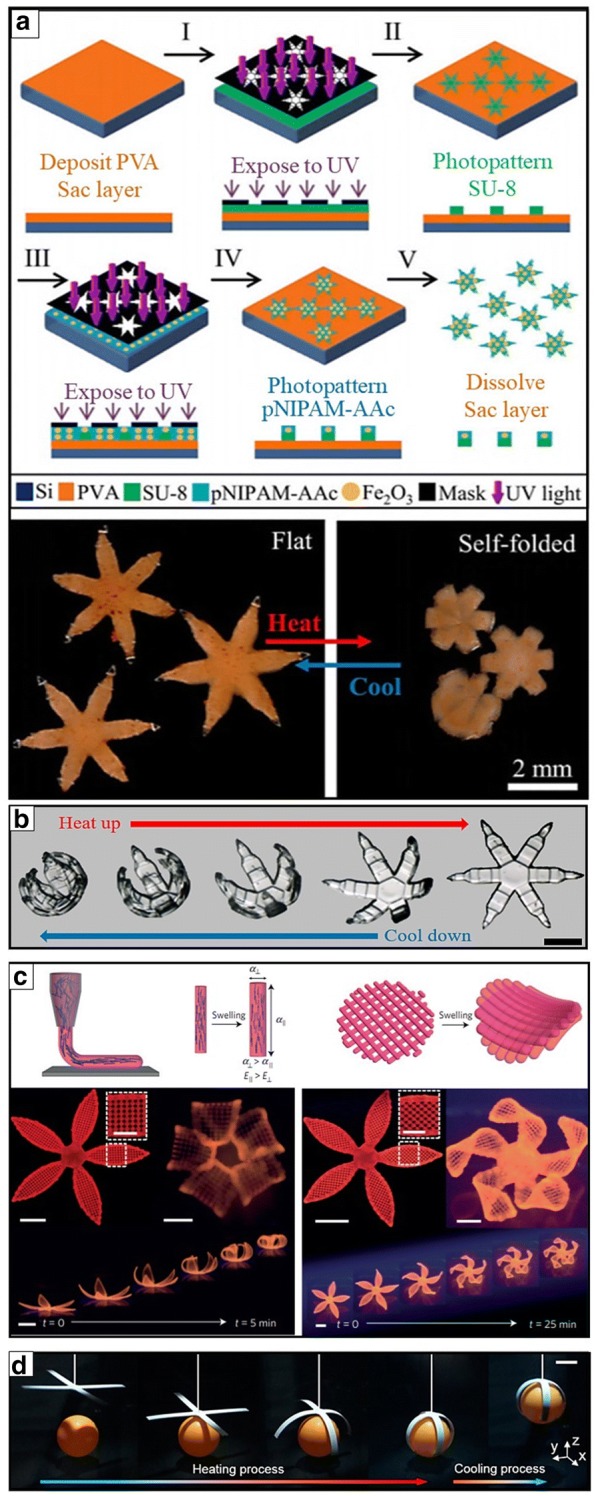
Two main photolithographic and 3D printing methods to fabricate stimuli responsive soft grippers. **a** Process flow of photolithography to create stimuli responsive soft grippers with following two-step UV exposures. The photopatterned bilayer soft grippers that close on heating and open up on cooling reversibly (reproduced with permission [79]. Copyright 2018, IEEE). **b** Photolithographically patterned biodegradable soft grippers that respond to temperature change (reproduced with permission [83]. Copyright 2019, American Chemical Society). **c** 4D printing that creates thermally responsive shape morphing flower shaped grippers composed of cellulose fibrils alignments programmed hydrogel composite ink (reproduced with permission [93]. Copyright 2016, Springer Nature). **d** 3D printed shape morphing soft grippers that pick-and-place a light ball (reproduced with permission [25]. Copyright 2018, American Chemical Society)

After tailoring stimuli responsive materials, fabrication methods have to be organized to realize stimuli-responsive 3D devices. In fabricating stimuli-responsive 3D devices, diverse methodological strategies, including direct manual assembly, printing, molding, top–down lithography, and bottom–up synthesis from macro to nano scales, have been proposed [4, 5, 34, 77, 78]. Despite the advances of these high-resolution and high-throughput technologies, the fabrication and assembly of stimuli-responsive 3D devices are still highly challenging. In patterning complex 3D stimuli-responsive soft robots, multistep fabrication and integration are required for more precise and functional systems. In this respect, the miniaturized and untethered stimuli-responsive soft grippers can be manufactured by utilizing combined self-folding and photolithographic strategies that guide a 2D thin film to a 3D curved, folded, or rolled shape transformation without any manual control [4, 5, 9, 10, 13, 26, 36, 39, 54, 64, 79–82]. Layering, printing, and direct casting are the generally widely used methods for constructing stimuli-responsive soft grippers [4]. Among several methodologies, two-step layering using photolithography is primarily selected to fabricate a broad range of shape transformable soft grippers. The shape-transformable thin bilayer structures patterned by two-step layering are mainly composed of swelling (active) and non-swelling (passive) layers [4]. These stimuli responsive thin bilayers can exhibit heterogeneous swelling that converts 2D thin films to 3D self-folded, -curved, or -rolled structures in response to external cues. Ionov et al. suggested several NIPAM-based shape morphing structures by using bilayer thin films and shapes [18, 39, 54, 80–82]. They specifically designed bilayer thin film structures composed of a thermoresponsive pNIPAM-based active layer and poly (methylmethacrylate-co-benzophenone acrylate) P(MMA-BA) or hydrophobic polycaprolactone (PCL)-based nonreactive passive layer. Gracias et al. also proposed several photolithographic processes to construct multifunctional complex structures that transform into 3D curved, folded, or rolled shapes in response to external thermal trigger [10, 13, 26, 36, 79, 83–88]. Serial aligning and patterning steps were strongly required to fabricate these multilayer or multidomain systems. They photopatterned stimuli-responsive soft grippers through a series of precise multilayer alignments (Fig. 4a) [79]. In addition, during the multi-photopatterning process, hydrogels easily adhere to the surfaces of untreated glass substrates and photomasks. They developed feasible protocols for the appropriate surface treatment of a sacrificial layered substrate and a chrome mask chamber to obtain selective adhesion of hydrogels during photopolymerization (Fig. 4b) [83].

In addition, 3D printing techniques have garnered significant attention for patterning 3D structures by a direct layer-by-layer process [89]. 3D printing enables the printing of various materials ranging from plastics [90] to softer gels [91] and even cell-cultured soft materials [92]. Four-dimensional (4D) printing has recently been highlighted as an innovative new technology [78, 93–96]. The combination of stimuli-responsive materials and the 4D printing technique has offered another new route to design 3D structures that change shapes over time with an appropriate stimulus. Gladman et al. demonstrated gripper morphologies generated by biomimetic 4D printing (Fig. 4c) [93]. They introduced a 4D printing technique using a programmable stimuli-responsive hydrogel composite ink to control the alignment of cellulose fibrils that adjust the anisotropic stiffness and swelling strain. The 4D printed thermally responsive biomimetic flower-shaped grippers were actuated through a temperature change-driven swelling process. Wang et al. also proposed shape-programmable polyester (PE) polymer–paper bilayer composites manufactured by 3D printing (Fig. 4d) [25]. They suggested a thermally responsive 3D-printed gripper that could pick-and-place a ball according to heating and cooling processes.

## Applications

### Soft actuators (tethered or untethered)

**Fig. 5:**
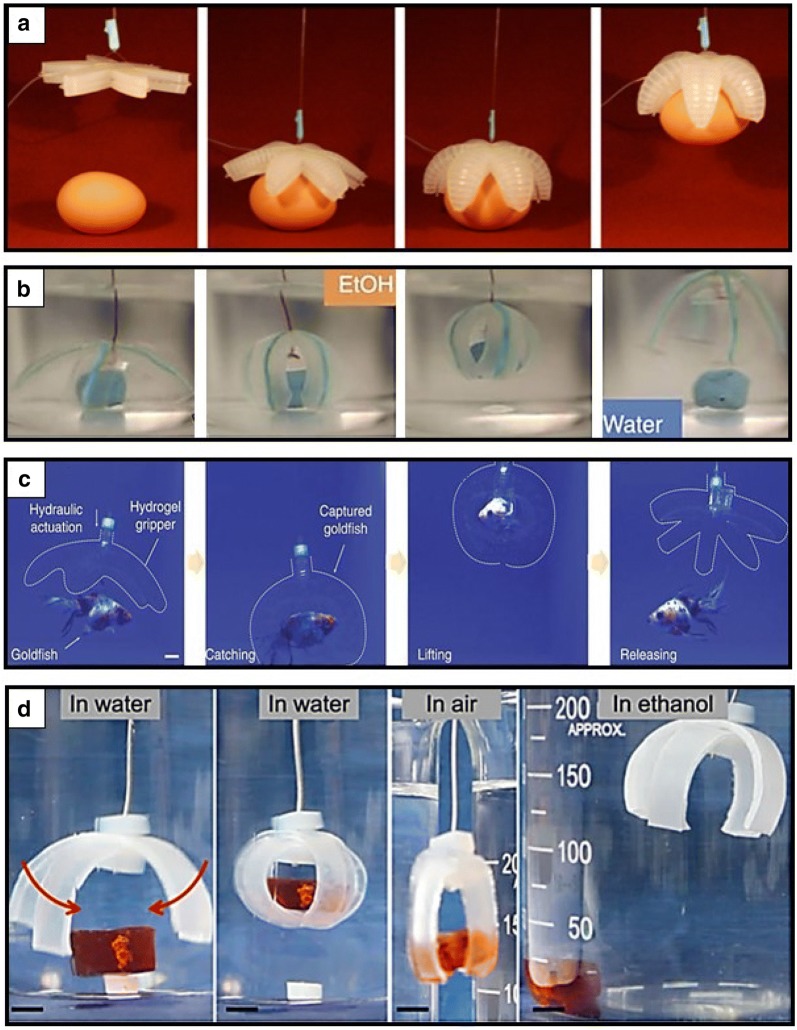
Tethered soft grippers as smart actuators or manipulators. **a** Pneumatically driven tethered soft gripping robot that provides a range of complex motions by curling upwards or downwards controlled by expansion and contraction in elastomeric structures (reproduced with permission [15]. Copyright 2011, Wiley–VCH). **b** X-shaped ion-printed tethered electrical assistant soft gripper that presents rapid grasp a target in ethanol and releases it in water reversibly (reproduced with permission [99]. Copyright 2013, Springer Nature). **c** Optically and sonically camouflageable hydraulic hydrogel gripping robot that holds and releases a live goldfish noninvasively (reproduced with permission [100]. Adapted with permission under the terms of the Creative Commons Attribution Non Commercial License 4.0 license. Copyright 2017, The Authors). **d** Stimuli responsive ion dip dyeing and transfer printed tough hydrogel-based soft gripper that can fold to grip a target in water and release it in ethanol reversibly (reproduced with permission [101]. Copyright 2016, Wiley–VCH)

Innovative methodologies, including photolithography and 3D printing, offer opportunities to create multiscale advanced functional soft machines, such as biomimetic actuators, drug delivery capsules, optoelectrical sensors, microsurgical devices, bioinspired tubulars, and artificial organs [34]. Although still in the conceptual stage, stimuli-responsive grippers have been extensively validated as smart actuators or manipulators, flexible sensors, mobile medicine, and surgical biopsy tools that are closely related to their shape programmability [4, 35, 38, 39, 97, 98]. Hitherto, a promising application of stimuli-responsive soft grippers is a smart actuator or a manipulator that can grip and release objects according to the stimuli on–off process. When designing soft grippers, tethered or untethered can be selected under a specific purpose. For example, tethered pneumatic-driven flexible silicon elastomer-based soft grippers can maintain precise actuation control (Fig. 5a) [15]. This new type of embedded pneumatic network systems can provide feasible and large amplitude actuations by pressurizing the embedded channels inside elastomeric soft grippers. In particular, the gripping motion requires pressure to permit a rapid actuation that leads to a range of complex motions by curling upwards or downwards. This pneumatic-driven soft gripper suggests new soft robotic areas of research using organic materials to noninvasively grip diverse large and heavy objects.

Another feasible method to control the actuation of hydrogel-based soft grippers is using tethered electrical assistance (Fig. 5b) [99]. Palleau et al. described an ionoprinted tethered electrical stimulus-driven soft gripper that rapidly actuates through mechanical strain coupled with electrically directed binding and different shrinkage. An X-shaped soft gripper was organized using sodium polyacrylate gel, which could gently manipulate an object not only in liquid, but also in air. This gripper was suspended with a wire to extract a polydimethylsiloxane target in ethanol and release it in water reversibly. This type of soft gripper exhibits a programmable temporal and spatial shape deformation as a new class of soft actuators. Meanwhile, Yuk et al. developed an optically and sonically camouflageable hydraulic soft transparent gripper that could noninvasively hold and release a live goldfish (Fig. 5c) [100]. They demonstrated its robustness and functionality by operating multiple actuation cycles. They also showed that hydraulic soft grippers could grip objects at a high speed of less than 1 s response time and a high force over 1 N compared to general osmotic hydrogel actuators. In addition, Peng et al. manufactured tethered programmable and complex shape-deformable tough hydrogel grippers by ion dip-dyeing and transfer printing (Fig. 5d) [101]. By selectively printing appropriate patterns on one-dimensional gel strips, a 3D stimuli responsive soft gripping robot was actuated after swelling the ion-patterned domains in water. This swelling-deformable stimulus-responsive ion-printed hydrogels can be used as a tethered soft gripper that grips (folding) a target in water and releases (unfolding) it in ethanol reversibly.

**Fig. 6:**
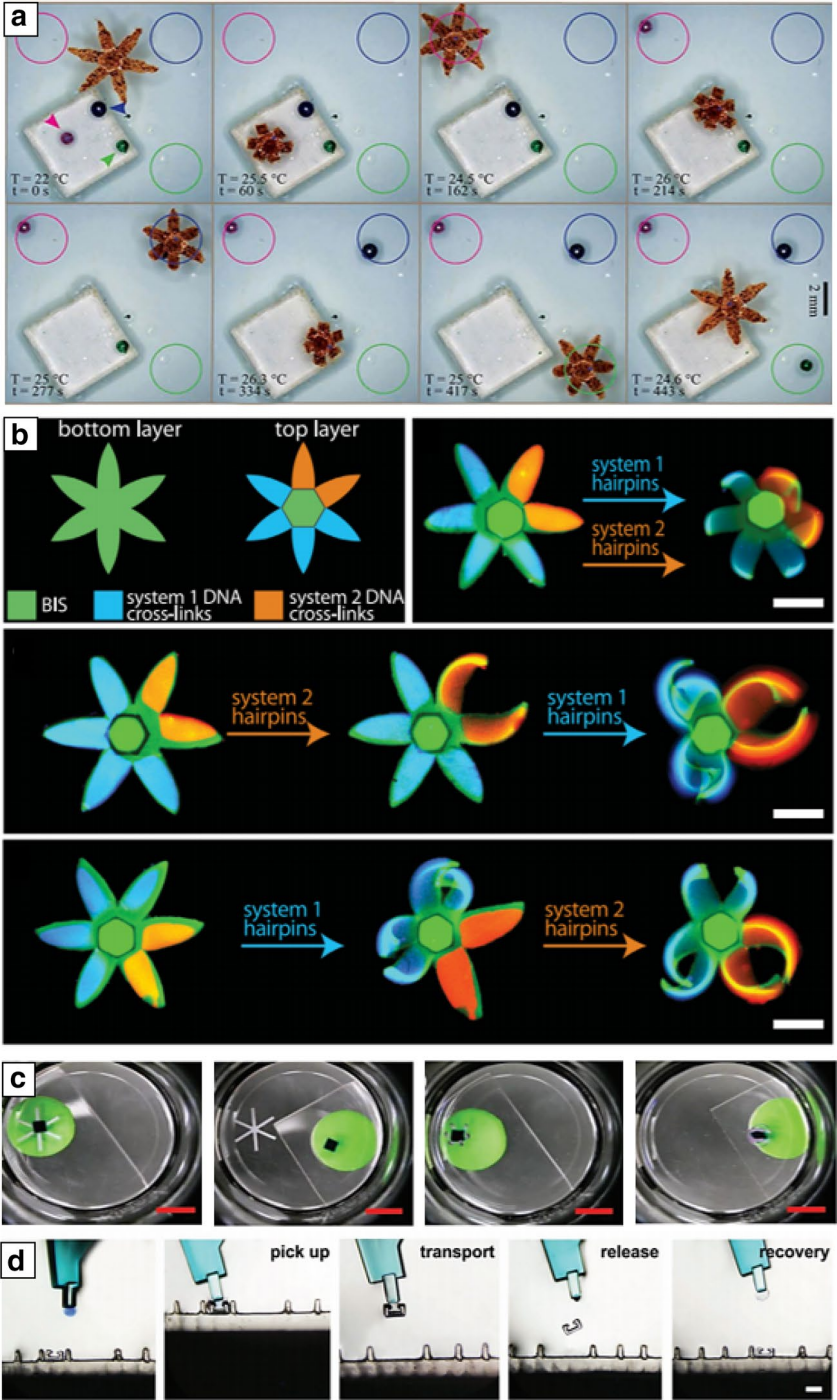
Untethered soft grippers as smart actuators or manipulators. **a** Thermomagnetically responsive untethered soft gripper that detects and sorts differently colored beads in the respectively colored drop areas autonomously (reproduced with permission [57]. Adapted with permission under the terms of the Creative Commons Attribution Non Commercial License 4.0 license. Copyright 2017, The Authors). **b** Shape programmable DNA-crosslinked untethered soft gripper that presents shape morphing in response to external programmed DNA hairpin sequences (reproduced with permission [102]. Copyright 2017, AAAS). **c** Bifurcation mismatch strain driven shape transformation of stimuli responsive soft gripper that has no hinges in a thin bilayer structure (reproduced with permission [8]. Copyright 2018, Wiley–VCH). **d** Demonstration of the universal pick up, transport, release, and recovery process of a soft robotic microgripper (reproduced with permission [21]. Copyright 2018, Wiley–VCH)

Another class of soft actuators include untethered hydrogel-based grippers. The untethered miniaturized small-scale soft robots can provide less-invasive approaches in a dynamically cluttered environment. Tetherless stimuli-responsive soft grippers can particularly be utilized as a new class of reconfigurable smart appliances that can be operated in a precisely controlled manner. For example, an untethered thermomagnetically responsive soft gripper could detect and sort different-colored beads then drop them in the corresponding colored target zones (Fig. 6a) [57]. The stimuli-responsive soft gripper demonstrated intelligent pick-and-place tasks under autonomously programmed dual magnetic and thermal triggers in unstructured aqueous environments without any wired or tethered systems. In addition, the Gracias group recently suggested smart DNA sequence-responsive bilayer hydrogel actuators by assembling two poly(acrylamide) hydrogel layers crosslinked with different DNA molecules (Fig. 6b) [102]. They created highly sensitive multi-domain DNA crosslinked grippers that could fold either simultaneously or sequentially through the external input of the programmed reactant DNA molecules in an aqueous environment [102].

Furthermore, Abdullah et al. proposed a stimuli-responsive shape morphing soft gripper composed of bilayer thin films with no hinges (Fig. 6c) [8]. They demonstrated the fundamental relationship of the bifurcation mismatch strain between layers of hingeless soft grippers and how it generated mechanical shape transformation through thermoresponsive shape morphing. Their demonstration of a finite element model and experimental validation suggested the applicable diverse array of gripping systems. In addition, an untethered soft gripper is valid for miniaturized microscale soft gripping robots. Jia et al. demonstrated a wide range of microscopic object transportations using a soft gripper, called the universal soft robotic microgripper (Fig. 6d) [21]. They proved the feasibility of the gripping functions by several combinations of geometrical interlocking between an untethered soft gripper and an object using shape memory and thermally responsive microgel structures. Furthermore, their untethered soft gripper was developed to conduct a series of pick up, transport, release, and recovery processes with no active visual or force feedback.

### Biological medicine

**Fig. 7:**
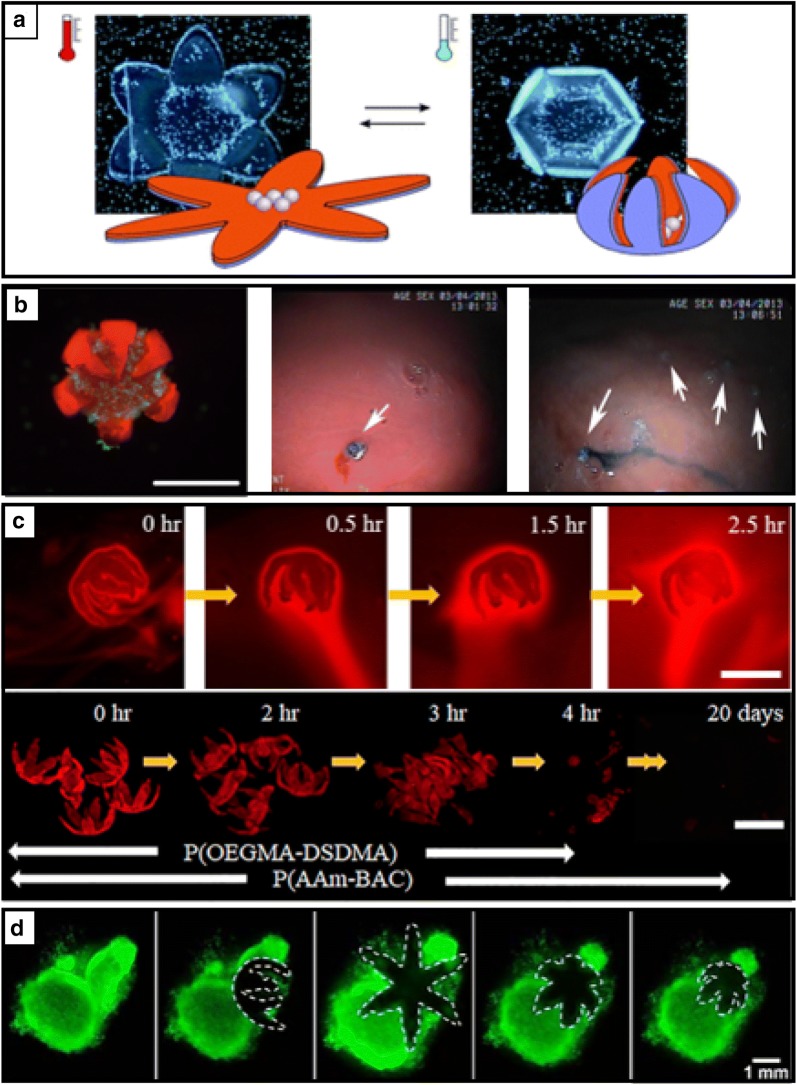
Stimuli responsive soft grippers for biomedical drug delivery or surgical biopsy. **a** Thermoresponsive self-folding microgrippers that can capture yeast cells by the deswelling induced contraction in the thermoresponsive layer that results in bending of the soft gripper at low temperature (reproduced with permission [18]. Copyright 2011, The Royal Society of Chemistry). **b** Therapeutic soft gripper that can grasp a clump of cells and demonstrate the endoscopic in vivo food dye delivery test to porcine stomach (reproduced with permission [10]. Copyright 2014, Wiley–VCH). **c** Biodegradable thermomagnetically responsive soft gripper for drug delivery (reproduced with permission [83]. Copyright 2018, American Chemical Society). **d** Self-folding thermo-magnetically responsive soft gripper that can capture and excision of cells from a live fibroblast clump (reproduced with permission [9]. Copyright 2015, American Chemical Society)

Stimuli-responsive micro robots have also addressed the key possible biomedical applications of advanced drug delivery and surgical biopsy tools. First, stimuli-responsive untethered hydrogels are advantageous for drug delivery. Ionov et al. suggested a thermoresponsive self-folding star-shaped bilayer composed of biodegradable hydrophobic PCL and pNIPAM-ABP (Fig. 7a) [18]. They demonstrated the reversible process of encapsulating and releasing yeast cells inside the self-folding capsules in response to a temperature signal. This thermally responsive soft gripper can be potentially utilized in drug delivery. Moreover, yeast cell-absorbed smart soft materials suggest the possibilities of cell-loaded 3D scaffolds for tissue engineering. The Gracias group developed therapeutic soft grippers (i.e., theragrippers) loaded with drugs that change shapes at the physiological body temperature using thermally responsive pNIPAM-based hydrogels (Fig. 7b) [10]. They developed three differently drug-loaded smart theragrippers to prove the feasibility of drug delivery by quantified experimental-drug releasing profiles and kinetic analysis. In addition, they proved the futuristic functionality of effective drug delivery in clinical conditions using untethered small-scale stimuli-responsive soft grippers that demonstrated endoscopic in vivo food dye delivery to porcine stomach. They also designed new biodegradable thin bilayer grippers composed of thermally responsive high-swelling poly(oligoethylene glycol methyl ether methacrylate-bis(2-methacryloyl) oxyethyl disulfide), P(OEGMA-DSDMA) and low-swelling poly(acrylamide-N,N′-bis(acyloyl)cystamine) P(AAm-BAC) (Fig. 7c) [83]. They particularly highlighted the potentials of intelligent drug delivery soft robots that can administrate drugs at target areas and degrade with no retrieving steps after drug release.

### Surgery

Miniaturized and untethered stimuli-responsive soft grippers have been proposed as attractive less-invasive surgical biopsy tools (Fig. 7b and d) [9, 10, 21, 83]. Current medical tools and techniques have been developed toward minimally invasive approaches. However, most of the dominant developments of medical instruments and techniques still include various wired or tethered systems that limit access to hard-to-reach areas inside a body. Even untethered biomedical devices, such as diagnostic image scanners [103] or biopsy tools [104], are relatively large because of the systematic integration of several functional parts, including batteries, sensors, and cameras, which results in another geometric limitation for scale downsizing. To resolve these restrictions, stimuli-responsive soft micro robots can be utilized as a new class of less-invasive intelligent soft machines. In this respect, Malachowski et al. demonstrated self-folding thermo-magnetically responsive pNIPAM-based grippers that could grasp a clump of cells under flow, which displayed the possibility of grasping mucosal tissue in the gastrointestinal tract (Fig. 7b) [39]. In addition, Breger et al. introduced a high-modulus (16 MPa) and stiff segmented polymer (i.e., PPF) to offer sufficient strength, such that a thermo-magnetically responsive untethered soft gripper could capture and excise cells from a live fibroblast clump (Fig. 7d) [9]. These examples highlight the potential application of shape-changeable untethered small-scale soft robots for intelligent less-invasive surgical biopsies.

## Conclusion

In summary, stimuli-responsive soft grippers have significantly attracted attention because of their promising applications in soft robotics, biomimetics, and biomedical engineering. Stimuli-responsive soft robots undergo programmable shape morphing in response to external environmental cues. Stimuli responsive materials, NIPAM-based hydrogels, liquid crystalline soft materials, and elastomers were preferentially considered to implement smart shape-deformable soft robots. Many innovative methodological strategies to construct gripper-shaped soft robots have specifically been developed over the past few decades. Photolithography or 3D printing is widely used in designing 3D shape-morphing soft grippers because of its scalability and manufacturability. A spatially controlled actuation response of approximately 10 × 30 μm in size of a stimuli-responsive NIPAM-based soft actuator was recently created using a two-photon absorption of focused light [105]. The development of this innovative two-photon lithographic strategy suggests the possibilities of the emergence of nanoscale stimuli-responsive soft grippers in the near future.

In addition, one of the most challenging points for stimuli-responsive soft grippers is the improvement of the response speed with the sensitivity feedback. The actuation of stimuli-responsive soft robots generally remains at a level of slow reactant behavior. Abnormal or nonlinear effects, such as sudden buckling or snapping of small-scale soft robots, are rarely explored. Another important challenging point is to develop soft grippers with tunable physical properties to delicately grip objects without damaging the gripper or the object itself. Many mechanical and/or physical modeling recently provided insights for understanding the shape morphing and stiffness of stimuli-responsive soft robots [8, 9, 61, 102, 106]. The shape change prediction by mechanical and/or chemical computational modeling of soft robots can facilitate the manufacture of soft robots possessing the proper softness to avoid target damage.

The precise navigation and transportation of soft grippers into target areas as well as the function of soft grippers is another big challenge, particularly, at deep in vivo locations for clinical drug delivery or biopsy. Since the human body is opaque, vision-based feedback and tracking of miniaturized soft robots are impossible. In order to overcome this limitation, recently, the ultrasound image feedbacks coupled gradient magnetic fields have been shown to be suitable for accurate control of the directionality of untethered soft robots for automated pick-and-place [57]. However, in order to realize autonomous in vivo navigation and transportation model of untethered soft robots, various unexplored magnetic resonance, ultrasound, or even near-infrared radiation guidance systems have to be developed in animal models at the near future. In conclusion, the innovative hybrid of material selections and synthesis, 3D manufacturing methods, and precisely controllable operation systems have to be developed in parallel to realize the multifunctionality, multi-responsible sensitivity, and highly sensitive feedback of new prospective soft gripping robots.

## Data Availability

Data sharing is not applicable to this article as no datasets were generated during the current study (review article).
